# Recent Advances in MXene-Based Electrochemical Sensors

**DOI:** 10.3390/bios15020107

**Published:** 2025-02-13

**Authors:** Ziyi Zhao, Jiayi Cao, Boyu Zhu, Xinru Li, Lin Zhou, Bin Su

**Affiliations:** Institute of Analytical Chemistry, Department of Chemistry, Zhejiang University, Hangzhou 310058, China; 22337054@zju.edu.cn (Z.Z.); 3200100159@zju.edu.cn (J.C.); 12337013@zju.edu.cn (B.Z.); 3160102072@zju.edu.cn (X.L.); 15988321707@163.com (L.Z.)

**Keywords:** MXene, two-dimensional nanomaterial, electrochemical sensor, electrochemical performance, application scenarios

## Abstract

MXene is a new family of two-dimensional nanomaterials with outstanding electrical conductivity, tunable structure, biocompatibility, and a large surface area. Thanks to these unique physicochemical properties, MXene has been used for constructing electrochemical sensors (MECSens) with excellent performance. In particular, the abundant surface termination of MXene can contribute to greatly enhancing the analytical sensitivity and selectivity of MECSens. Recently, MECSens have been widely applied in many fields including clinical diagnosis, infectious disease surveillance, and food security. However, not all MXene materials are suitable for building electrochemical sensors. In this article, we present an overview of different MECSens that have been developed so far. We begin with a short summary of the preparation and characterization of MECSens. Subsequently, the electrochemical performance, detection strategies, and application scenarios of MECSens are classified and briefly discussed. The article ends with a short conclusion and future perspectives. We hope this article will be helpful for designing and constructing MECSens with outstanding activity for electrochemical analysis.

## 1. Introduction

Nanomaterials have emerged as a cornerstone of modern materials science due to their unique physicochemical properties and wide application potential. Among the various nanomaterials, two-dimensional (2D) materials (e.g., graphene [1,2,3], transition metal dichalcogenides [4,5], black phosphorous [6,7], MXene [8,9,10], etc.) have gained significant attention thanks to their high surface area, tunable electronic structure, and outstanding mechanical strength, which make them ideal candidates for numerous advanced sensor technologies [11,12]. MXene, as a family of 2D early transition metal carbides, nitrides, and carbonitrides, is obtained by selectively etching the aluminum or silicon element in the MAX phase [13,14,15]. Since the first type of MXene (Ti_3_C_2_T_x_) was prepared by Gogotsi et al. in 2011, more than 40 types of MXene materials have been synthesized in the past 15 years [16]. Because of its unique physicochemical properties, MXene has found widespread applications in numerous fields, such as sensing, catalysis, energy storage, environmental remediation, and biomedical technology [17,18,19,20,21].

**Scheme 1:**
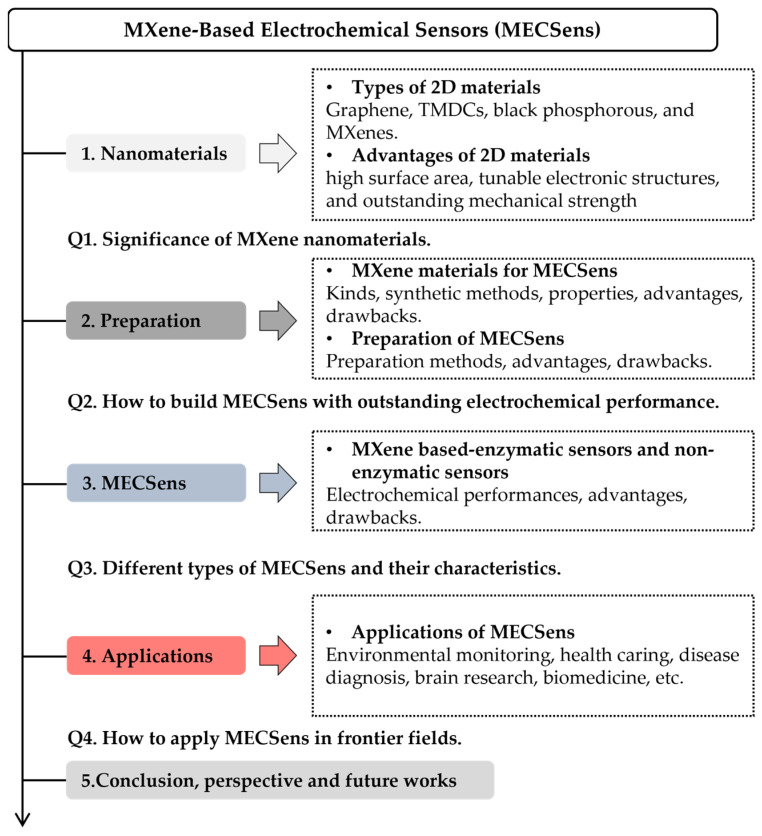
The major topics discussed in this review article.

It merits particular attention that, very different from other 2D materials, MXene offers enormous promise for the electrochemical sensing application due to its outstanding electrical conductivity, hydrophilicity, tunable structure, biocompatibility, and large surface area [22,23,24]. MXene can improve surface conductivity and electron mobility and catalyze electrochemical reactions, eventually enhancing the sensitivity, selectivity, stability, and reproducibility of electrochemical sensors (ECSens) [25,26,27]. Although a large number of MXene-based electrochemical sensors (MECSens) have been fabricated for detecting neurotransmitters, heavy metal ions, drugs, reactive oxygen species, glucose, and biomarkers, the development of MECSens is still in the early stage [28,29,30]. There are rarely review articles to guide the MECSens’ design and construction. This review article aims to discuss different kinds of MECSens and present an overview of their recent advances (Scheme 1). We shall first discuss, from the fundamental viewpoint, the physicochemical property of MXene materials and feasibility of their application in MECSens. In subsequent sections, the performance, detection strategies, and application scenarios of MECSens will be summarized. The summary will elaborate on the advantages and drawbacks of different MECSens. Finally, the article will end with the conclusion and future perspectives on the work of designing MECSens to meet the requirements of complex ECSens in frontier fields of biomedical, cell, in vivo, and brain research.

## 2. Preparation and Fabrication of MECSens

Although numerous MXene materials have been synthesized, not all of them are suitable for constructing ECSens. The preparation process of MXene materials usually involve steps of etching, delamination, functionalization, etc. The preparation process basically determines MXene’s physicochemical property [31]. The physicochemical property is a key factor for preparing MECSens with outstanding electrochemical performance, because their electrical conductivity, hydrophilicity, thermal conductivity, and chemical composition greatly impact the surface charge and mass transfer, further influencing the sensitivity, selectivity, and stability of MECSens. In this section, the synthetic methods, advantages, and drawbacks of different MXene materials for MECSens, as well as the preparation methods of MECSens, are briefly presented.

### 2.1. Synthesis of MXene Materials for MECSens

**Figure 1:**
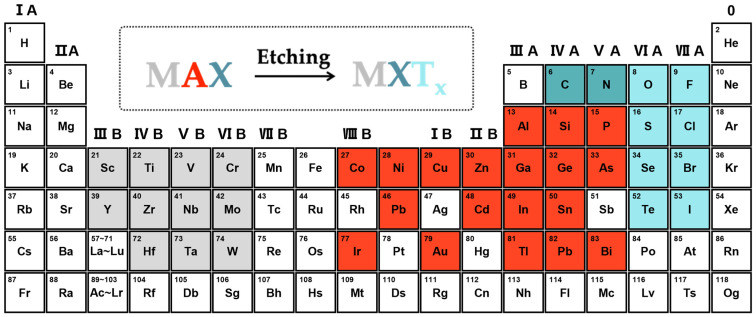
The MAX phases and MXene materials.

The general chemical formula of MXene is MXT_x_, where M, X, and T represent the transition metal (e.g., titanium, vanadium, molybdenum, tantalum, etc.), carbon/nitrogen, and surface termination (e.g., hydroxyl, oxygen, fluorine amino-group, etc.), respectively (Figure 1) [32,33,34]. The MXene materials are synthesized by etching the layers of “A” from the bulky MAX phases via the wet chemical method, the Lewis acid molten salt etching method, or the electrochemical method. Importantly, different synthetic methods will bring unique physicochemical properties to the MXene materials, which determine whether they are suitable for MECSens [35,36].

#### 2.1.1. F-Containing Etching Method

**Figure 2:**
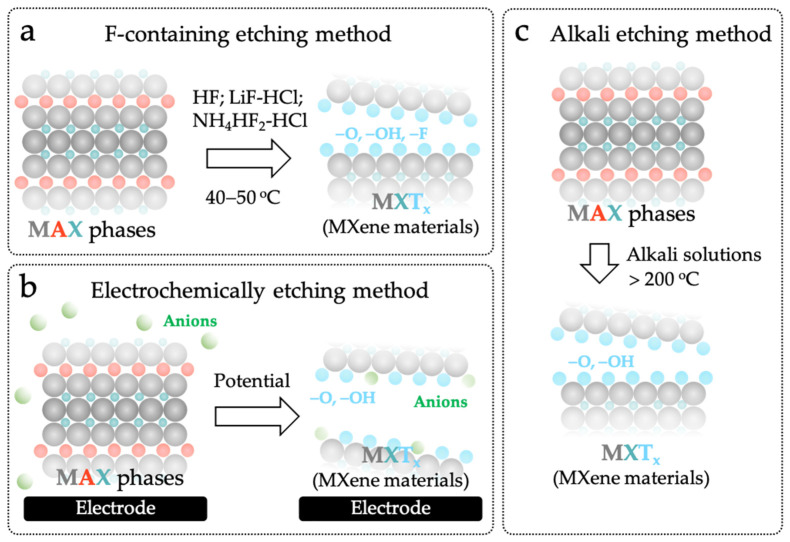
Different methods for synthesizing MXene materials: (**a**) F-containing etching method; (**b**) Electrochemically etching method; (**c**) Alkali etching method.

As shown in Figure 2a, F-containing etching method is the most commonly used wet chemical method. The etchants, including hydrofluoric acid (HF), ammonium bifluoride (NH_4_HF_2_), or lithium fluoride-hydrochloric acid (LiF-HCl), are used to etch the Al layer in the MAX phases [33,37,38,39]. Although the F-containing etching method needs toxic and hazardous etchants, it is very simple and effective. For example, two-dimensional Ti_3_C_2_T_x_ with an accordion-like morphology can be easily obtained by treating Ti_3_AlC_2_ in an HF solution at 40–50 °C for several hours [40,41,42,43]. However, this method will result in -O, -OH, and -F surface terminations on MXene nanosheets [44,45,46]. In particular, the -F termination can decrease the electrical conductivity and increase the hydrophobicity of the MXene materials, which greatly hinders and slows down the charge transfer, causing the phenomenologically small current, large non-faradaic capacitive current and poor analytical sensitivity (Table 1) [47,48]. Therefore, the application of MXene materials obtained by the F-containing etching method in electrochemical sensing is still limited.

#### 2.1.2. Non-F-Containing Etching Method

To overcome the limitations of F-containing etching method, a lot of non-F-containing etching methods have been developed to prepare “electrochemically friendly” MXene materials [49,50]. Among them, the electrochemically etching method is the most moderate one. As illustrated in Figure 2b, the layers of “A” from the bulky MAX phases can be oxidized and removed to obtain MXene materials by biasing a high positive potential on the MAX phases under room temperature [51,52]. More importantly, the chemical property of MXene materials can be well controlled by adjusting the potential composition of the electrolyte solution and the etching time [32]. The M-X bonds are broken, and the anions in the electrolyte solution are doped into MXene nanosheets during the etching, yielding controllable surface terminations [50]. For example, Green et al. electrochemically etched Ti_2_AlC in 2 M HCl aqueous solution at a potential of +0.5 V to obtain Ti_2_CT_x_ MXene [51]. When extending the etching time from 1 day to 14 days, the contents of O and Cl gradually increase from 8.30% to 24.68% and from 0.00% to 0.84%, respectively. Que et al. found that the doping of anions can be remarkably inhibited while electrochemically etching the MAX phases in the alkali solution, even at a very high potential (+1–5 V) [53]. However, the drawback of electrochemical etching method is also obvious. The formation of by-products, such as the three-layer structure composed of MAX core and carbide-derived carbon, will block the active sites of MAX phase thus impacting the etching efficiency and quality (Table 1) [15,54].

Alkali etching method is another non-F-containing etching method (Figure 2c). Briefly, the “A” layers can be completely removed by treating the MAX phases in highly concentrated alkali solutions (such as 6 mol L^−^^1^ of NaOH and KOH solutions) at a high temperature (>200 °C) [49]. In comparison with those prepared by the F-containing etching and electrochemically etching methods, the MXene materials obtained by this method only contain -O and -OH surface terminations, and possess excellent hydrophilicity, electrical conductivity, electrochemical catalytic property and stability, which meet all requirements for building MECSens with outstanding electrochemical performance [55].

### 2.2. Preparation of MECSens

**Figure 3:**
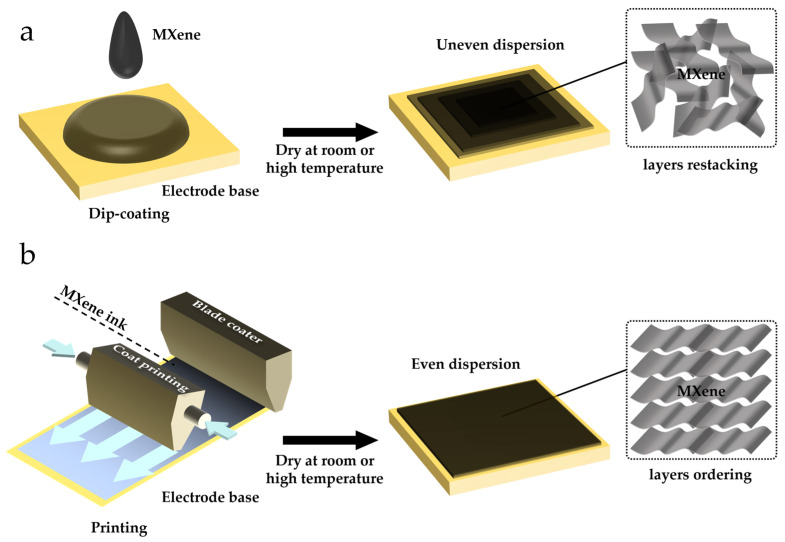
The methods used for preparing MECSens: (**a**) dip-coating method; (**b**) printing method.

As shown in Figure 3, various strategies, such as dip-coating and screen-printing, have been developed for constructing MECSens [56,57,58]. The main challenge is how to enhance the MECSens’ stability [20,59,60]. First, due to the poor dispersity and agglomeration of MXene materials, it is hard to uniformly modify them on the electrode surface. Second, the exfoliation of MXene materials from ECSens cannot be easily avoided [26,36]. In order to solve these issues, the traditional strategies have been improved, and new approaches for preparing MECSens have also been developed. In this section, the advantages, drawbacks, and development of strategies for the preparation of MECSens are presented.

#### 2.2.1. Dip-Coating Method

Dip-coating is the most convenient method to prepare MECSens (Figure 3a). First, the MXene solution is dip-coated on the substrate electrode. Then, the electrode is dried under room or high temperature condition. However, due to the poor dispersity and agglomeration (π-π and electrostatic interactions) of MXene materials, they can hardly be coated uniformly on the electrode surface and may easily be exfoliated from ECSens [61,62]. The MECSens obtained by dip-coating method usually display poor electrochemical performance and insufficient stability, so they are not suitable for electrochemical analysis in a long-time scale [63].

In order to improve the stability of MECSens, cationic/anionic surfactants and alkylates are intercalated into the interlayers of MXene nanosheets to enlarge the interlayer spacing thus inhibiting the restacking and improving the dispersity [41,64,65]. Tang et al. intercalated cetyltrimethylammonium bromide (CTAB) into the interlayers of Ti_3_C_2_T_x_ nanosheets (CTAB@Ti_3_C_2_T_x_) via electrostatic adsorption. In this way, CTAB@Ti_3_C_2_T_x_ could be completely and uniformly coated on the electrode surface, showing a smooth morphology. In contrast, after the dip-coating of Ti_3_C_2_T_x_ on the electrode surface, bulk Ti_3_C_2_T_x_ blocks were randomly stacked together. The electrochemical performance and stability of a CTAB@Ti_3_C_2_T_x_-coated electrode are much better than those of a Ti_3_C_2_T_x_-coated electrode [66]. Talapin et al. chemically modified alkylamines on MXene nanosheets by reacting halogen-terminated MXene with deprotonated organic amines, which strictly controlled the interlayer spacing of MXene nanosheets with a sub-nanometer precision. The MXene materials obtained could be well dispersed in aqueous solutions for 14 days; meanwhile, the restacking of the MXene nanosheets was also effectively inhibited [67]. It should be noticed that, although these strategies are very effective, the non-conductive hydrophobic surfactants and alkylates may hinder and slow down the charge transfer and mass transport, resulting in large capacitance, phenomenologically small current, poor analytical sensitivity and, eventually, significant loss of the electrochemical activity of MECSens [31,67].

#### 2.2.2. Printing Method

The printing of MXene ink on flexible substrates is another method for preparing MECSens [68,69]. MXene ink is usually prepared by mixing MXene with adhesives and additives, which can be easily printed on substrates by 3D printing, screen-printing, inkjet printing, writing, or stamping [61,70,71,72]. In comparison with dip-coating, the printed MXene materials are more uniform, and exfoliation from the substrate can be also effectively avoided. Due to the uneven stacking of MXene materials, the MXene nanosheets cannot entirely contact with the electrode surface, which decreases the conductivity of MECSens [73,74]. Fortunately, it can be easily overcome by adjusting the ink formulation. For example, the bulky MXene in ink can be removed by vacuum filtration. And additives including sodium ascorbate, sodium oxalate, sodium citrate and sodium phosphate can be added [75,76].

Numerous high-quality MXene inks have been prepared, which have been successfully used in supercapacitor, energy storage, and energy conversion [72,77,78]. However, their applications in electrochemical sensing are still rarely reported because, similar to surfactants and alkylates, adhesives and additives in the ink can deactivate and passivate MECSens [79,80]. Therefore, the adhesive- and additive-free MXene inks have been prepared by dispersing the less aggressively etched MXene materials in organic solvents. The electrochemical performance of prepared MECSens is much superior to that of ECSens printed by the traditional MXene inks [81,82,83]. In general, comparing with the dip-coating method, the printing method is the better approach for preparing MECSens.

#### 2.2.3. Other Methods

Apart from above two methods, other methods including the sol–gel method, vacuum-assisted filtration, and hot-pressing have also been employed to prepare MECSens [84,85,86,87,88]. However, these methods are complicated. They often involve a series of complex and time-consuming steps [89,90]. The complex pre-treatments of MXene materials and substrate electrodes are usually required [91,92]. Therefore, these methods are not usually used to prepare MECSens.

### 2.3. Characterization Techniques of MXene

Different characterization techniques have been used to comprehensively investigate the structural, chemical, and electrochemical properties of MXene materials (Table 2). For example, field emission scanning electron microscopy (FESEM) can be used to investigate the surface morphology and structural features of MXene [93]; X-ray photoelectron spectroscopy (XPS) is an indispensable tool for analyzing the surface chemistry and elemental composition of MXene [94]; X-ray diffraction (XRD) is an effective technique for investigating the crystalline structure, interlayer spacing, and etching degree of MXene [95]; Infrared (IR) spectroscopy can identify surface functional groups and the chemical bonds in MXenes [96]; Raman spectroscopy provides detailed information about the vibrational modes of lattice, interlayer interactions, and structural defects [97]. In a word, these methods provide critical insights into the morphology, composition, surface termination, and electronic structure of MXenes, enabling their optimization for specific applications of MECSens.

## 3. Different Types of MECSens and Their Applications

ECSens are categorized into two types, namely enzymatic and non-enzymatic ECSens [98,99]. Due to their excellent specificity, selectivity, sensitivity, and biocompatibility, enzymatic ECSens have been widely utilized in electrochemical sensing [100,101,102]. However, the electrochemical performance of enzymatic ECSens often suffer from the exfoliation and degradation of enzymes [103]. In enzymatic MECSens, the MXene materials are usually used for enzyme immobilization via physical adsorption, entrapment, or covalent bonding, which can effectively slow down the exfoliation and degradation of enzymes, thus enhancing the stability of the sensors [103]. For example, Gu et al. constructed a 3D porous Ti_3_C_2_T_x_-based hybrid film for glucose oxidase (GOx) immobilization. The GOx entered the pores of Ti_3_C_2_T_x_, increasing the immobilization stability while retaining the activity of GOx. The prepared MECSen showed good ability toward glucose sensing, and the current response to glucose has no obvious changes after repeated use for 300 times [104]. Fan et al. reported a facile method to covalently bond enzymes on the surface of MXene nanosheets through the coordination interaction with aminosilane ligand spacers [105]. In comparison with unimmobilized enzymes, immobilized enzymes exhibit outstanding stability and reusability, which are very important for the practical application of MECSens. Alshareef et al. synthetized large-area Ti_3_C_2_T_x_ MXene films by the minimally intensive layer delamination method to facilely immobilize enzymes for designing wearable MECSens [106]. The large-area MXene films greatly enhance the stability of the wearable ECSens during physical activities and over extended periods. However, it should be noted that the role of MXene materials in enzymatic MECSens has no difference with other nanomaterials (such as graphene, covalent organic frameworks, metal organic frameworks, etc.) and thus the advantage of MXene is not obvious.

Nevertheless, the unique layered structure of MXene nanosheets provides abundant catalytic active sites on their surface. Thus, the MXene materials with structural tunability and diversity offer enormous promise for building non-enzymatic MECSens for the sensitive and selective detection of neurotransmitters, oxygen, glucose, reactive oxygen species, drugs, metal ions, and organic pollutants (Table 3). In particular, by ingeniously modulating the structures and properties of MXene materials (as discussed in Section 2), the electrochemical performance of non-enzymatic MECSens can be precisely controlled and enhanced, which is hardly achieved using other nanomaterials. In this section, the advantages, challenges, and development of non-enzymatic MECSens, as well as their practical applications in electrochemical analysis, are presented.

### 3.1. Glucose

**Figure 4:**
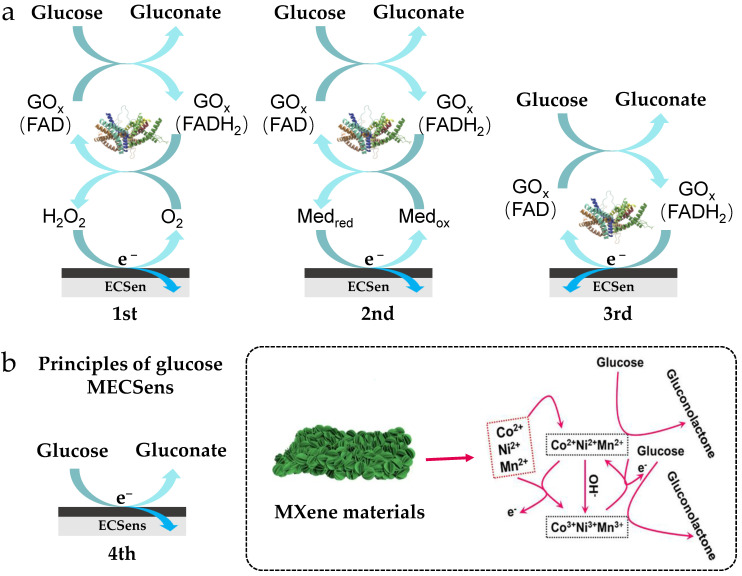
The enzymatic glucose ECSens (**a**) and nonenzymatic glucose MECSen (**b**). Reproduced from Ref. [109], with the permission from Elsevier, Copyright 2024.

Glucose is one of the most common organic molecules in the natural world, which serves as an important energy source. According to the difference in electron transfer mechanisms, enzymatic glucose ECSens are divided into three types. The non-enzymatic glucose ECSens are called as the fourth type glucose sensor (Figure 4a), in which glucose is directly oxidized on the MECSens, and the current produced by the oxidation reaction is proportional to the concentration of glucose. Recently, MXene materials have been used to build the fourth type glucose sensor. Glucose in blood, sweat, tears, saliva, or interstitial fluid has been successfully detected by MECSens, providing great convenience of self-monitoring for diabetics.

In non-enzymatic glucose MECSens, the MXene materials not only act as active interfacial sites for glucose oxidation but also mediate the rapid electron transfer. Compared with the enzymatic glucose MECSens, the non-enzymatic glucose MECSens display high sensitivity, low limit of detection, wide linear range, and fast response time. The composites of Cu_x_O, ZnO, Ni, Co, and Pt with MXene have also been prepared to modify MECSens, which are very sensitive to glucose, yielding a low limit of detection (<1 μM), a high sensitivity (>100 μA mM^−1^ cm^−2^), and a wide linear range (~0.001–30 mM) (Figure 4b. Therefore, the non-enzymatic glucose MECSens hold promise to replace the traditional glucometer. For example, Song et al. and Zhou et al. reported a series of Cu_x_O-MXene coated carbon-based electrodes for enzyme-free detection of glucose in sweat, blood, and urine [114,115]. These glucose sensors exhibited good sensitivity, selectivity, and long-term stability, which could stably work in real samples (blood and serum) for weeks without any loss of electrochemical performance. Choi et al. synthesized a Ni, Co lnd Mn decorated-Ti_3_C_2_T_x_ MXene material to act as the electrode material for non-enzymatic glucose sensing [109]. The hierarchical structure among Ni, Co, Mn, and MXene can efficiently prevent agglomeration and restacking of MXene nanosheets, ensuring the exposure of active interfacial sites for glucose oxidation. Therefore, the electrochemical performance and repeatability of these glucose sensors are much better than others.

Moreover, the wearable glucose MECSens also have been developed for continuous glucose monitoring. As shown in Figure 4b, the wearable glucose MECSens can be prepared by printing the MXene ink on the flexible substrates. These wearable MECSens were directly stuck on the tissue or organs, and then the glucose concentration in the interstitial fluid, sweat, or tear was detected. Using these MECSens, the change in glucose concentration with the replenishment and consumption of energy by the body during exercise, diet, or work was successfully recorded. However, limited by the difficulty in manufacture (discussed in Section 2.2), the wearable non-enzymatic glucose MECSens are still rarely reported.

### 3.2. Ascorbic Acid, Dopamine, and Uric Acid

Ascorbic acid (AA), dopamine (DA) and uric acid (UA) widely exist in the neuron, urinary, and endocrine systems, which are closely relate to Parkinson’s disease, Alzheimer’s disease and /renal diseases/. However, the molecular structure and oxidation potential of AA, DA, and UA are very similar. They generate comparable and overlapped electrochemical signals, hindering the precise quantification of each of them. Because of the tunable surface charge and chemical structure, the MXene materials can enhance the selectivity of MECSens to the AA, DA, and UA.

**Figure 5:**
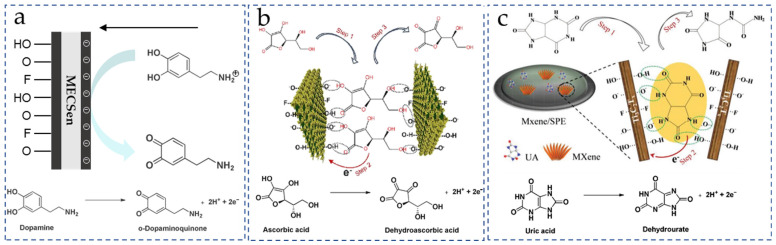
Sensing mechanisms of non-enzymatic DA (**a**), AA (**b**), and UA (**c**) MECSens. Reproduced from Ref. [135], with the permission from Elsevier, Copyright 2023. Reproduced from Ref. [140], with the permission from Wiley, Copyright 2018.

First, oxygen-containing functional groups such as -O and -OH on MXene nanosheets provide the MECSens with the negatively charged surface, which can repel the mass transport of anionic AA and UA via the electrostatic repulsion (Figure 5a). In this case, the selective detection of DA in the mixture of AA, DA and UA is easily achieved. Thus, numerous MECSens have been prepared for detecting DA in human serum, hydrochloride injection, and urine [119,120,121]. Moreover, the electrostatic interaction between negatively charged MXene nanosheets and positively charged DA at neutral pH contributes a lot to the analytical sensitivity of the MECSens. Taking these advantages, MECSens have been used to monitor subtle changes in DA level in biological systems. For example, both the cellular level of DA released from neuronal cells and the increase in DA concentration in human sweat were successfully measured using the DA MECSens.

Second, the selectivity and electrocatalytic reaction mechanisms of UA and AA on the MECSens are mainly associated with the functional groups (-O, -OH and -F) and the interlayer structure of MXene nanosheets. As illustrated in Figure 5b,c, only UA and AA are adsorbed on the interlayer of MXene by the π-π interaction and by forming hydrogen bonds with functional groups, which can thus be electrochemically oxidized and detected by MECSens. Furthermore, the π-π interaction between the MXene nanosheets and six-member heterocyclic aromatic compounds is more stable than five-member ring systems [137]. The adsorption energy of UA in the interlayer structure is much stronger than that of AA, leading to the higher Gibbs free energy of electrochemical oxidation. So, the MECSens possess higher sensitivity and oxidation potential towards UA.

According to these mechanisms, AA, DA and UA can be simultaneously analyzed by adjusting the surface charge, chemical structure, and interlayer structure of the MXene modified MECSens. Among all MXene materials, the Ti_3_C_2_T_x_ MXene is the most ideal candidate and the Ti_3_C_2_T_x_-modified electrodes have been used to detect AA, DA, and UA simultaneously. The variations of AA, DA, and UA in sweat, urine and other real samples can be monitored.

### 3.3. Hydrogen Peroxide

Hydrogen peroxide (H_2_O_2_) is one of the reactive oxygen species, which occupies a very important position in neuron activity, brain function, and neurodegeneration disorders. However, due to its low concentration, the quantitative analysis of H_2_O_2_ is very difficult. So far, although a large amount of enzymatic H_2_O_2_ ECSens has been developed, the poor reproducibility, low durability and high cost still hinder their pratical applications. In recent decades, numerous non-enzymatic H_2_O_2_ ECSens have been prepared to replace the traditional enzymatic ECSens, such as Pt, graphene, hemoglobin, and copper based ECSens. These materials are used because of their electrocatalytic activity toward the oxidation of or reduction in H_2_O_2_. However, due to high overpotential and electron transport kinetic limitation, these non-enzymatic H_2_O_2_ ECSens still cannot meet the requirement of practical applications.

**Figure 6:**
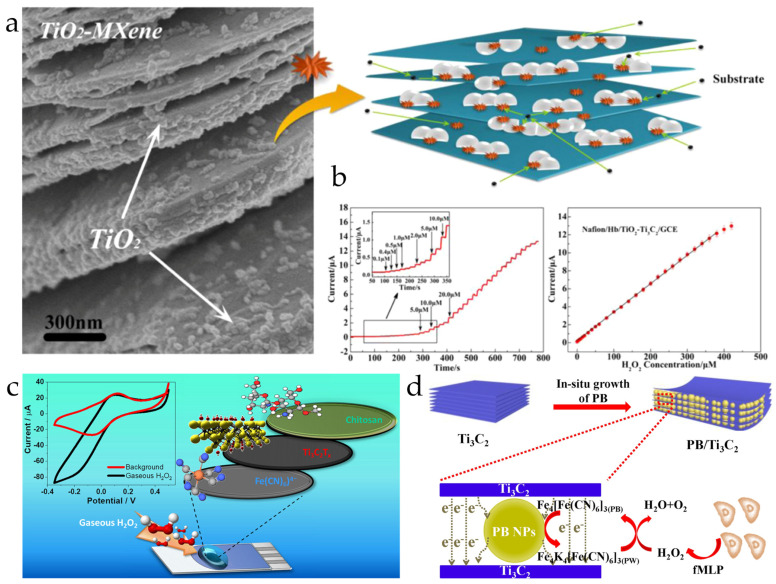
(**a**) The microscopic morphology and schematic illustration of TiO_2_-Ti_3_C_2_ MXene nanocomposite. Reproduced from Ref. [145], with the permission from Elsevier, Copyright 2015. (**b**) The chronoamperometric current response and linear range obtained with TiO_2_-Ti_3_C_2_ MXene nanocomposite-modified GCE. (**c**) Schematic diagram of preparing MECSen for the detection of H_2_O_2_ Reproduced from Ref. [147], with the permission from American Society of Chemistry, Copyright 2023. (**d**) Schematic illustration of the MECSens for detecting H_2_O_2_ released from cells. Reproduced from Ref. [144], with the permission from Elsevier, Copyright 2020.

Because of excellent electrical conductivity and high surface activity, MXene materials are used to improve the electrochemical performance of non-enzymatic H_2_O_2_ ECSens by enhancing the electron transfer and reducing the overpotential. Yang et al. used a Ti_3_C_2_T_x_ MXene nanocomposite to increase the sensitivity of hemoglobin-modified glassy carbon electrode (GCE) to H_2_O_2_. The Ti_3_C_2_T_x_ MXene nanocomposite not only increases the electron transfer rate between hemoglobin and electrode but also favors the immobilization of more hemoglobin to increase the collision between redox hemoglobin and electrode. This MECSen shows a good response to H_2_O_2_, yielding a detection limit of 14 nM and a linear range of 0.1–380 μM, which are lower and wider than those obtained with GCE merely modified with hemoglobin (Figure 6a) [145]. Hočevar et al. found Ti_3_C_2_T_x_ MXene on the ferrocyanide-modified screen-printed carbon electrode for the detection of trace H_2_O_2_ (<4 ppbv). In the presence of H_2_O_2_, Fe(CN)_6_^4−^ is chemically oxidized to Fe(CN)_6_^3−^ and then immediately reduced back to Fe(CN)_6_^4−^ at the electrode surface, leading to the increase and decrease in the reduction and oxidation currents with an increase in H_2_O_2_ concentration. Because MXene can catalyze the electrochemical conversion of Fe(CN)_6_^4−^/Fe(CN)_6_^3−^, the detection potential is decreased by 100 mV and the sensitivity increased by 3-fold (Figure 6b) [147]. The same effect of MXene materials has been found for other non-enzymatic H_2_O_2_ ECSens, including Cu-based metal organic framework/MXene-modified electrode, Prussian blue/MXene-modified electrode, and Fe_2_O_3_ composite-modified electrode.

The outstanding electrochemical performance of H_2_O_2_ MECSens allows for the real-time detection of H_2_O_2_ in living cells (Figure 6c). H_2_O_2_ released from human cervical cancer cells (HeLa) under *N*-formylmethionyl-leucyl-phenylalanine (fMLP) and KCl stimulations is detected [144]. However, the drawback of H_2_O_2_ MECSens cannot be ignored. The MXene materials are easily oxidized at a high concentration of H_2_O_2_. Therefore, H_2_O_2_ MECSens are not suitable to detect highly concentrated H_2_O_2_ in industrial wastewater, detergents, disinfectants, and etc.

### 3.4. Heavy Metal Ions

**Figure 7:**
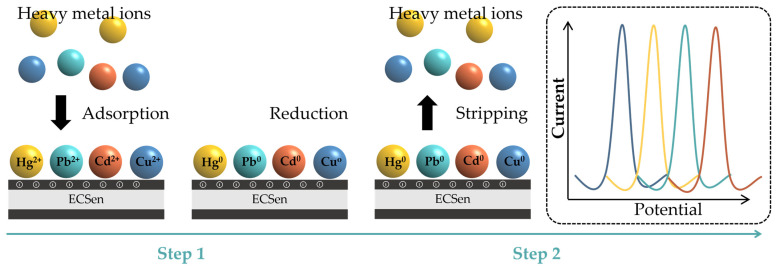
Electrochemical detection of heavy metal ions by ECSens.

Heavy metal ions, such as Fe^3+^, Cd^2+^, Cu^2+^, Pb^2+^, Hg^2+^, Co^2+^, Mn^2+^, Zn^2+^, etc., are the most frequently detected environmental pollutants, which heavily threaten human health because their accumulation in the body can cause organ damage and numerous diseases. Recently, ECSens have been regarded as ideal tools for the sensitive and selective detection of heavy metal ions. The analytical procedure involves two steps (Figure 7). First, heavy metal ions are adsorbed and electrochemically reduced on the ECSens’s surface to form corresponding zero-valent metals at a constant negative potential (pre-concentration step). Second, the zero-valent metals are oxidized back to heavy metal ions by anodic stripping voltammetry. The produced currents at different potentials are proportional to the concentrations of different heavy metal ions. The first pre-concentration step determines the analytical sensitivity. The MXene materials have the ability to enhance the efficiency of pre-concentration step via the electrostatic interaction between heavy metal ions and negatively charged MXene nanosheets. In this case, MECsens display outstanding analytical performance. Different heavy metal ions with several ppb level in water samples, foods, and fruits can be successfully detected by MECSens.

Furthermore, by adjusting the chemical property of MXene, the selectivity of MECSens to different heavy metal ions can be well controlled. For example, LiF-HCl-etched Ti_3_C_2_T_x_ MXene prefers to adsorb Cu^2+^, and thus the Ti_3_C_2_T_x_-modified MECSen is only highly sensitive to Cu^2+^. The limit of detection is lower than 0.095 nM [154]. To ingeniously increase the content of the -O and -OH groups from 13.37 to 33.20% by treating Ti_3_C_2_T_x_ MXene in the alkaline solution, the current responses obtained with MECSens to Cd^2+^ and Pb^2+^ can be greatly enhanced [151]. In addition, the amino group can promote the adsorption of Hg^2+^. Because the direct amino-functionalization of MXene materials is very difficult, amino-functionalized 2D materials (such as g-C_3_N_4_ and N-dropped graphene) can be mixed with MXene materials to enhance the performance of MECSens for Hg^2+^ detection. Han’s group modified GCE with a layered N-doped carbon/Ti_3_C_2_T_x_ MXene composite, which showed a high sensitivity to Hg^2+^, yielding a low limit of detection of 0.056 μM and thus allowing for the quantitative analysis of trace Hg^2+^ in natural water and foods [153]. Shahid et al. used the Ti_3_C_2_T_x_/g-C_3_N_4_ nanocomposite to modify GCE for ultrasensitive electrochemical detection of Hg^2+^, achieving a low limit of detection at 0.26 nM, which is much lower than the permissible limit recommended by the WHO guidelines [157]. Therefore, by adjusting the functional groups on the MXene nanosheets, the selectivity of MECSens is tunable for detecting various heavy metals.

## 4. Conclusions

In this review, we summarized the latest advances in MXene-based electrochemical sensors. Very different from other nanomaterial-based ECSens, the strength of MECSens is very obvious. Briefly, the electrochemical performance of MECSens can be precisely controlled and enhanced by ingeniously modulating the structures and properties of MXene materials. Thanks to the outstanding electrochemical performance, MECSens have been widely applied in many fields. Numerous analytes including glucose, uric acid, dopamine, ascorbic acid, hydrogen peroxide, and heavy metals can be sensitively and selectively detected by MECSens. However, the weakness of MECSens cannot be ignored. Due to poor dispersity, agglomeration, and exfoliation of MXene materials, MECSens still cannot overcome the challenge of long-time electrochemical analysis well, although they can satisfy the short-time electrochemical analysis. The practical applications of MECSens for real-time health monitoring, brain research, and in vivo electrochemical analysis still face serious challenges. In these bio-conditions, the MECSens will also suffer from more severe interferences, such as biofouling, immune reactions, and biological degradation. The improvements in antibiofouling ability, biocompatibility, anti-biodegradation, in vivo stability, and selectivity of MXene materials and MECSens should be taken into consideration. Moreover, wearable and implantable ECSens will be the major focus of academia and industry in the future. The biocompatible MXene is the ideal material to prepare these sensors. Wearable or implantable ECSens can be used to detect various biomarkers in real time, providing more comprehensive monitoring and better disease management for patients.

## Figures and Tables

**Table 1:** Advantages and disadvantages of different methods for synthesizing MXene materials.

Method	Advantages	Drawbacks	Suitable
F-containing etching method	-Most commonly used method -Very simple and effective --O, -OH and -F surface terminations	-Very toxic and hazardous -Poor electrical conductivity -Poor electrochemical property	** 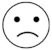 **
Electrochemically etching method	-Most moderate method -Controllable	-Low efficiency -A large amount of by-products	** 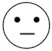 **
Alkali etching method	-High efficiency -High hydrophilicity -High electrical conductivity -High stability	-High temperature	** 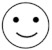 **

**Table 2:** Different characterization techniques.

Technique	Purposes	Key Observations	Applications
FESEM	Surface morphology and structure	Accordion-like layered morphology, delaminated nanosheets, surface roughness	Evaluates surface area and active site exposure
XPS	Surface chemistry and elemental analysis	Quantification of -F, -O, -OH terminations, detection of dopants or functional groups	Optimizes chemical composition for specific sensor applications
XRD	Crystalline structure and interlayer spacing	Shift in (002) peaks indicating successful etching or intercalation, layer stacking information	Confirms etching and delamination efficiency
IR Spectroscopy	Surface functional groups and bonding configurations	-Detection of -O, -OH, and -F terminations via characteristic vibrational modes (e.g., broad -OH at 3200–3500 cm^−1^, -F at 1100–1200 cm^−1^) -Evidence of intercalation and chemical modifications	-Evaluates hydrophilicity and ion transport properties -Assesses compatibility for electrochemical sensors and energy storage devices
Raman Spectroscopy	Analyze lattice vibrations, defects, and interlayer interactions	-Identification of M-X bonds (e.g., Ti-C at 200–800 cm^−1^) -Identification of structural defects and disorder in terms of changes in peak intensity/position -Detection of interlayer interactions and shear modes	-Monitors material quality and electron transfer efficiency -Tracks real-time changes during electrochemical reactions -Guides optimization of MXene for sensing and catalytic applications

**Table 3:** Different non-enzymatic MECSens.

	MECSen	Analyte	Method	Performance	Ref
1.	GCE/Ti_3_C_2_T_x_-Co@NC	Glucose	Chronoamperometry (CA)	Detection limit: 66.8 nM Detection range: 0.5–100 μM Detection sensitivity: 370 μA mM^−1^ cm^−2^	[107]
2.	GCE/Ti_3_C_2_T_x_/NiCo-LDH	Glucose	CA	Detection limit: 0.53 μM Detection range: 2–4096 μM Detection sensitivity: 64.75 μA mM^−1^ cm^−2^	[108]
3.	GCE/Ti_3_C_2_T_x_/NiMn-LDH NCs	Glucose	CA	Detection limit: 0.24 μM Detection range: 10 μM–900 M	[109]
4.	Cu_2_O/Ti_3_C_2_T_x_/rGO	Glucose	CA	Detection limit: 1.1 μM Detection range: 0.1 μM–40 mM	[110]
5.	Cu/Cu_2_O/C/Ti_3_C_2_T_x_	Glucose	CA	Detection limit: 0.103 μM Detection range: 1 μM–26.3 mM	[111]
6.	GCE/Ti_3_C_2_T_x_/Cu_2_O	Glucose	CA	Detection limit: 2.83 μM Detection range: 0.01 μM–30 mM Detection sensitivity: 11.061 μA mM^−1^ cm^−2^	[112]
7.	Pt/CH/Ti_3_C_2_T_x/_Nafion	Glucose	CA	Detection range: 0–8 mM	[113]
8.	Au@CuO/LIG/V_2_CT_x_	Glucose	CA	Detection limit: 1.8 μM Detection range: 0.005–5 mM	[114]
9.	CFP/TC Ti_3_C_2_T_x_/Cu_x_O	Glucose	CA	Detection limit: 0.065 μM Detection range: 1 μM–4.655 mM 5.155–16.155 mM Detection sensitivity: 361 μA mM^−1^ cm^−2^ 133 μA mM^−1^ cm^−2^	[115]
10.	Cu_2_O/Ti_3_C_2_T_x_/GCE	Glucose	CA	Detection limit: 1.96 μM Detection range: 0.004–13.3 mM 15.3–28.4 mM Detection sensitivity: 430 μA mM^−1^ cm^−2^ 240 μA mM^−1^ cm^−2^	[116]
11.	ZnO/Ti_3_C_2_T_x_/Nafion/Au	DA	CA	Detection limit: 0.076 μM Detection range: 0.1–1200 μM Detection sensitivity: 96 nA μM^−1^	[117]
12.	GCE/Ti_3_C_2_T_x_/G-MWCNTs/ZnO	DA	Differential Pulse Voltammetry (DPV)	Detection limit: 3.3 nM Detection range: 0.1–30 μM Detection sensitivity: 16A M^−1^	[118]
13.	GCE/S-MXene/HG	DA	DPV	Detection limit: 0.06 μM Detection range: 0.5–50 μM	[119]
14.	ITO/MXene/DODA	DA	DPV	Detection limit: 72.2 nM Detection range: 0–50 μM Detection sensitivity: -	[120]
15.	3DE/MQDs	DA	CV	Detection limit: 3 nM Detection range: 0.01–20 μM Detection sensitivity: -	[121]
16.	CC/Nb_2_CT_x_@MoS_2_	DA	CA	Detection limit: 0.23 fM Detection range: 1 fM–100 μM	[122]
17.	GCE/Ti_3_C_2_T_x_/Nafion	DA	CA	Detection limit: 3 nM Detection range: 0.015–10 μM	[123]
18.	Ti_3_C_2_T_x_/ERHG/GCE	DA	CA	Detection limit: 1.1 μM Detection range: 0.1 μM–40 mM Detection sensitivity: -	[124]
19.	Pt/Ti_3_C_2_T_x_/PC-SPCEs	DA	DPV	Detection limit: 28 nM Detection range: 0.1–200 μM	[125]
20.	GCE/Nb_2_CT_x_/ZnS	DA	DPV	Detection limit: 1.39 μM Detection range: 0.09–0.82 mM Detection sensitivity: 12.1 μA μM^−1^	[126]
21.	SPE/CeO_2_/Ti_3_C_2_T_x_/ABSAG	DA	CA	Detection limit: 0.017 μM Detection range: 0.05–300 μM	[127]
22.	GCE/Ti_3_C_2_T_x_ nanosheets	AA	DPV	Detection limit: 2 nM Detection range: 0.005–300 μM	[128]
23.	GPE/Ti_3_C_2_T_x_/1-methyl imidazolium acetate	DA	CA	Detection limit: 702 nM Detection range: 10–2000 μM FA: Detection sensitivity: 9.61 μA mM^−1^ cm^−2^	[129]
24.	Ti_3_C_2_T_x_/CCY	DA	CA	Detection limit: 316 pM Detection range: 1 nM–1 μM	[130]
25.	GCE/MOF/Nb4C3Tx	DA	DPV	Detection limit: 0.2 nM Detection range: 1–100 nM Detection sensitivity: -	[131]
26.	PAA/MXene/PI	UA	DPV	Detection limit: 2.48 μM Detection range: 10–400 μM	[132]
27.	GCE/PyTS@Ti_3_C_2_T_x_	UA	DPV	Detection limit: 0.48 μM Detection range: 5–100 μM	[133]
28.	TiO_2_ NPs/Ti_3_C_2_T_x_/rGO	UA	DPV	Detection limit: 0.78 nM Detection range: 0.003–300 μM	[134]
29.	MoS_2_/Ti_3_C_2_T_x_	AA	CA	Detection limit: 4.2 μM Detection range: 50–250 μM Detection sensitivity: 54.6 nA μM^−1^	[135]
30.	GCE/AuNP@Ti_3_C_2_T_x_	UA, Folic acid (FA)	CA	Detection limit: UA: 11.5 nM FA: 6.2 nM Detection range: UA: 0.03–1520 μM FA: 0.03–1520 μM Detection sensitivity: UA: 0.53 μA mM^−1^ cm^−2^ FA: 0.494 μA mM^−1^ cm^−2^	[55]
31.	LIG/NiO/Ti_3_C_2_T_x_	AA, DA, UA	DPV	Detection limit: AA: 35 μM DA: 0.4 μM UA: 0.08 μM Detection range: AA: 20–2000 μM DA: 0.1–100 μM UA: 0.2–100 μM	[136]
32.	GCE/Ti_3_C_2_T_x_	AA, DA, UA	DPV	Detection limit: AA: 4.6 μM DA: 0.06 μM UA: 0.075 μM Detection range: AA: 100–1000 μM DA: 0.5–50 μM UA: 0.5–4 μM 100–1500 μM	[137]
33.	CFP/MXene/MoS_2_	AA, DA, UA	DPV	Detection limit: AA: 1.47 μM DA: 0.27 μM UA: 0.38 μM Detection range: AA: 10–3000 μM DA: 0.5–1000 μM UA: 0.5–1000 μM	[138]
34.	Au-Pd/MXene/LSG	AA, DA, UA	DPV	Detection limit: AA: 3 μM DA: 0.13 μM UA: 1.47 μM Detection range: AA: 10–1600 μM DA: 12–240 μM UA: 8–800 μM	[139]
35.	GCE/MXene/Cu^2+^	Creatinine (Cre), UA, Urea	DPV, Square wave voltammetry (SWV)	Detection limit: UA: 5 μM Cre: 1.2 μM Detection range: UA: 30–500 μM Urea: 0–3 mM μM Cre: 10–400 μM	[140]
36.	GCE/Ti_3_C_2_T_x_	H_2_O_2_	CA	Detection limit: 3.5 nM Detection sensitivity: 596 mA mM^−1^ cm^−2^	[141]
37.	GCE/Fe_2_O_3_/Ti_3_C_2_T_x_	H_2_O_2_	Cyclic voltammetry (CV)	Detection limit: 7.46 nM Detection range: 5–30 μM Detection sensitivity: 0.32 μA mM^−1^ cm^−2^	[142]
38.	GCE/Pt NPs/Ti_3_C_2_T_x_	H_2_O_2_	CA	Detection limit: 448 nM Detection range: 0.49–53.6 mM Detection sensitivity: 0.32 μA mM^−1^ cm^−2^	[143]
39.	GCE/PB NPs/Ti_3_C_2_T_x_	H_2_O_2_	CA	Detection limit: 0.2 μM Detection range: 0.6–254 μM	[144]
40.	GCE/Nafion/TiO_2_/Ti_3_C_2_T_x_	H_2_O_2_	CA	Detection limit: 14 nM Detection range: 0.1–380 μM Detection sensitivity: 447.3 μA mM^−1^ cm^−2^	[145]
41.	GCE/rGO/Ti_3_C_2_T_x_/MWCNTs	H_2_O_2_	CA	Detection limit: 0.3 μM Detection range: 1–60 μM 60 μM–9.77 mM Detection sensitivity: 235.2 μA mM^−1^ cm^−2^ 103.8 μA mM^−1^ cm^−2^	[146]
42.	SPCE/Ti_3_C_2_T_x_/CHI	H_2_O_2_	CA	Detection limit: 4 μg m^−3^ Detection range: 0.5–30 μg m^−3^	[147]
43.	GCE/HRP/Ti_3_C_2_T_x_/Nafion	H_2_O_2_	DPV	Detection limit: 1 μM Detection range: 5–8000 μM Detection sensitivity: -	[148]
44.	GCE/BiFeO_3_/Ti_3_C_2_T_x_	Pb^2+^	Differential pulse anodic stripping voltammetry (DPASV)	Detection limit: 0.0001 μg L^−1^ Detection range: 0.0003–1000 μg L^−1^	[149]
45.	GCE/alk-Ti_3_C_2_T_x_	Cd^2+^, Pb^2+^, Cu^2+^, Hg^2+^	Square wave anodic stripping voltammetry (SWASV)	Detection limit: Cd^2+^: 0.098 μM Pb^2+^: 0.041 μM Cu^2+^: 0.032 μM Hg^2+^: 0.130 μM	[150]
46.	GCE/Ti_3_C_2_T_x_@rGO/UiO-66-NH_2_	Cd^2+^, Pb^2+^	DPASV	Detection limit: Cd^2+^: 0.46 ppb Pb^2+^: 0.40 ppb Detection range: Cd^2+^: 2–800 ppb Pb^2+^: 1–600 ppb	[151]
47.	GCE/Ti_3_C_2_T_x_ NR	Cd^2+^	DPV	Detection limit: 0.94 nM Detection range: 0.005–3.0 μM	[152]
48.	GCE/N-doped Carbon/Ti_3_C_2_T_x_	Cu^2+^, Hg^2+^	SWV	Detection limit: Cu^2+^: 0.019 μM Hg^2+^: 0.056 μM Detection sensitivity: Cu^2+^: 114.54 μA mM^−1^ cm^−2^ Hg^2+^: 64.317 μA mM^−1^ cm^−2^	[153]
49.	GCE/cmc/Ti_3_C_2_T_x_	Cu^2+^	SWV	Detection limit: 0.095 nM Detection range: 1–10,000 nM	[154]
50.	GCE/Ti_3_C_2_T_x_ MNR@Au	Hg^2+^	DPV	Detection limit: 17 pM Detection range: 0.4 nM–2 μM	[155]
51.	GCE/CoPB/Ti_3_C_2_T_x_	Cu^2+^	SWASV	Detection limit: 0.97 nM Detection range: 1–200 nM	[156]
52.	GCE/Fe_3_O_4_/g-C_3_N_4_/Ti_3_C_2_T_x_	Zn^2+^, Cd^2+^, Pb^2+^, Cu^2+^, Hg^2+^	DPASV	Detection limit: Zn^2+^: 0.26 nM Cd^2+^: 0.21 nM Pb^2+^: 0.10 nM Cu^2+^: 0.11 nM Hg^2+^: 0.12 nM Detection range: 0.005–0.5 μM	[157]
53.	GCE/AuNPs/Ti_3_C_2_T_x_	Pb^2+^	SWV	Detection limit: 0.3 μM Detection range: 0.5–300 μM	[158]
54.	ITO/AuNPs@MBA/Ti_3_C_2_T_x_	Pb^2+^, Cu^2+^, Hg^2+^	DPASV	Detection limit: Cd^2+^: 0.07 μg L^−1^ Cu^2+^: 0.13 μg L^−1^ Hg^2+^: 0. 21 μg L^−1^ sensitivity: Cd^2+^: 1.157 μA μg^−1^ cm^−2^ Cu^2+^: 0.846 μA μg^−1^ cm^−2^ Hg^2+^: 0.799 μA μg^−1^ cm^−2^	[159]
55.	SPCE/3D melamine-doped GO/Ti_3_C_2_T_x_	Zn^2+^, Cd^2+^, Pb^2+^	DPASV	Detection limit: Zn^2+^: 0.48 μg L^−1^ Cd^2+^: 0.45 μg L^−1^ Pb^2+^: 0.29 μg L^−1^ Detection range: 3–900 μg L^−1^	[160]
56.	Au NPs/Ti_3_C_2_T_x_	Pb^2+^	Capacitance method	Detection limit: 0.43 pM Detection range: 0.01–7500 nM	[161]

## Data Availability

Not applicable.
